# Deep Learning Model Using Transfer Learning for Detecting Left Ventricular Systolic Dysfunction: Retrospective Algorithm Development and Validation Study

**DOI:** 10.2196/83127

**Published:** 2026-04-24

**Authors:** Sungjae Lee, Jung-Woo Son, Sung-Ai Kim, Min-Soo Ahn, Sang Jun Lee, Sang-Jin Han, Taehyun Joo, Yeongyeon Na, Sunghoon Joo, Hyun Jin Ahn, Mineok Chang, Yeha Lee, Young Jun Park

**Affiliations:** 1 VUNO Inc, Seoul, Republic of Korea; 2 Division of Cardiology, Department of Internal Medicine, Wonju Severance Christian Hospital, Yonsei University Wonju College of Medicine, Wonju, Republic of Korea; 3 Division of Cardiology, Department of Internal Medicine, Hallym Sacred Heart Hospital, Hallym University College of Medicine, Anyang, Republic of Korea; 4 Division of Cardiology, Department of Internal Medicine, Korea University Anam Hospital, Korea University College of Medicine, 73 Goryeodae-ro, Seongbuk-gu, Seoul, Republic of Korea, 82 2 720 5445

**Keywords:** left ventricular systolic dysfunction, deep learning, electrocardiogram, echocardiography, artificial intelligence

## Abstract

**Background:**

Artificial intelligence–augmented electrocardiogram (AI-ECG) models for detecting left ventricular systolic dysfunction (LVSD) often exhibit degraded performance in patients with comorbidities.

**Objective:**

This study aimed to introduce and validate a recalibration method using longitudinal patient data to enhance prediction accuracy and simulate its clinical utility for ongoing monitoring.

**Methods:**

We conducted a multicenter, retrospective cohort study using data from 2 hospitals in Korea. A dataset of paired transthoracic echocardiograms (TTEs) and electrocardiograms (ECGs) matched within a 2-week interval was constructed, separating pairs into baseline (first for each patient) and follow-up assessments. In addition to conventional supervised learning, we developed a patient-wise recalibration strategy that incorporated historical left ventricular ejection fraction measurements and prior AI-ECG outputs to adjust for future predictions, thus empirically mitigating confounding effects. Pretraining was also implemented to enhance the model’s performance.

**Results:**

The recalibrated 12-lead DeepECG LVSD model achieved an area under the receiver operating curve of 0.956 (95% CI 0.946‐0.965) for internal validation and 0.940 (95% CI 0.936‐0.945) for external validation of follow-up TTE-ECG pairs. The uncalibrated 12-lead DeepECG LVSD model also showed modest performance, with an area under the receiver operating curve of 0.953 (95% CI 0.941‐0.965) in the internal validation and 0.947 (95% CI 0.943‐0.951) in the external validation when tested on baseline TTE-ECG pairs. Recalibration yielded statistically significant improvements in the 12-lead DeepECG LVSD models (*P*<.001), with enhanced and more balanced performance across all clinical subgroups.

**Conclusions:**

Patient-wise recalibration improved accuracy and consistency across various comorbidities by mitigating performance degradation and bias. This broadens the application of AI-ECG for LVSD detection from low-risk screening to high-risk longitudinal monitoring.

## Introduction

### Overview

Left ventricular systolic dysfunction (LVSD) is a critical precursor of symptomatic heart failure (HF) and is associated with a substantially elevated risk of cardiovascular morbidity and mortality [1,2]. Despite the clinical benefits of early detection, including the timely initiation of guideline-directed medical therapy [3], LVSD is often underdiagnosed until overt symptoms emerge. This diagnostic delay is largely attributable to a lack of efficient and scalable screening tools applicable to the general population.

Conventional diagnostic modalities, such as echocardiography, cardiac magnetic resonance imaging, and biomarker assays, although accurate, are costly, time-consuming, and influenced by confounding clinical variables, such as age and renal function. As such, they are not ideally suited for widespread screening initiatives. By contrast, electrocardiogram (ECG) remains a more accessible and cost-effective tool for detecting cardiovascular diseases. In this context, early studies, including those by Attia et al [4-7], developed artificial intelligence–augmented electrocardiogram (AI-ECG) algorithms capable of screening for LVSD using ECG inputs. Several prospective observational [8-10] and interventional [11] studies have since sought to validate the clinical utility of these artificial intelligence (AI)-assisted diagnostic strategies.

In parallel, a paradigm shift in HF management has occurred [12,13], with increasing emphasis on identifying and managing patients in the early or at-risk stages. In this context, AI-based detection of LVSD using ECG may offer a more accessible alternative to echocardiography and provide benefits for this at-risk population. However, early AI studies have shown limited performance in certain subgroups, such as older individuals [14] and patients with atrial fibrillation (AFIB) [6,10,14,15], groups that are commonly encountered in clinical practice and substantially overlap with those at a higher risk of HF.

Thus, we developed a deep learning–based framework (ie, DeepECG LVSD) consisting of three sequential learning phases: (1) large-scale ECG pretraining using automatically generated labels, (2) supervised fine-tuning for LVSD detection using paired baseline transthoracic echocardiography-electrocardiogram (TTE-ECG) data, and (3) a patient-wise recalibration strategy that incorporates prior left ventricular ejection fraction (LVEF) measurements and historical AI-ECG outputs. We evaluated this framework using both internal and external validation cohorts, seeking to enable a more reliable and clinically adoptable application of AI-ECG in routine cardiac care.

### Model Overview

The proposed DeepECG LVSD framework takes electrocardiographic recordings as input and outputs a continuous risk score for LVSD. The model is developed using a 3-stage learning strategy comprising large-scale ECG pretraining with automatically generated labels, supervised fine-tuning for LVSD detection using paired baseline TTE-ECG data, and a patient-specific recalibration phase. The recalibration component leverages longitudinal patient information, including prior AI-ECG outputs and, when available, corresponding LVEF measurements, to adjust individual risk estimates and stabilize model behavior at a fixed decision threshold across heterogeneous clinical subgroups. To enhance applicability in settings where echocardiography is unavailable, we additionally evaluated a masked-LVEF variant that performs recalibration using serial ECG-derived information only. Full architectural details, training procedures, and evaluation protocols are provided in the Methods section.

## Methods

### Consent

We conducted a multicenter, retrospective cohort study at 2 university hospitals in Korea: Hallym Sacred Heart Hospital (Hospital A) and Wonju Severance Christian Hospital (Hospital B).

### Study Population and Data Acquisition

For model development and internal validation, we identified adult patients from Hospital A (n=259,943, January 2006 to December 2020) who underwent standard 12-lead ECG, including a subset of 59,433 patients who underwent transthoracic echocardiography (TTE). Adult patients from Hospital B (n=67,377; November 2007 to December 2018) who underwent both ECG and TTE were identified for external validation. Standard 10-second 12-lead ECGs recorded at 250 Hz or 500 Hz sampling rates using Marquette ECG machines and the MUSE software system (General Electric Healthcare) were extracted to construct the ECG dataset. For the TTE dataset, free-text TTE reports were parsed to extract echocardiographic parameters, including LVEF. Demographic variables (age and sex) and *ICD-10* (*International Statistical Classification of Diseases, Tenth Revision*) diagnosis codes were obtained from electronic medical records for characterization in baseline and subpopulation analyses. Records from the TTE and ECG datasets were paired to construct the TTE-ECG dataset. For each patient, TTE recordings were matched to the closest ECG acquired within a 14-day window period [4]. Patients may have had multiple eligible pairs that were further categorized based on whether the TTE-ECG pair represented the first match (baseline TTE-ECG pair) or a subsequent match (follow-up TTE-ECG pair).

For Hospital A, patients were included in the supervised fine-tuning if they underwent both TTE and ECG recordings within a 14-day window. Eligible TTE-ECG pairs were randomly assigned to the training, validation, and test sets in an 8:1:1 ratio and used during supervised training. If a patient had multiple eligible pairs, all the pairs from the same patient were assigned to the same split. ECGs from patients without any eligible TTE-ECGs were also randomly split into training, validation, and test sets and used in the pretraining phase, as described later.

### Definition of Key Variables

The target condition of our AI-ECG model was the presence of LVSD, defined as an LVEF of ≤40%. TTE reports may contain multiple LVEF values derived using different methods, including the Simpson method (biplane approach), M-mode (2D), and visual estimation by an examiner. When multiple LVEF values were reported, a single value was selected based on a predefined order of priority, that is, Simpson method, M-mode, and visual assessment. The selected LVEF value was then converted into binary labels indicating the presence (positive TTE-ECG pair if LVEF was ≤40%) or absence (negative TTE-ECG pair if LVEF was >40%) of an LVSD.

### Development of the AI-ECG Algorithm

**Figure 1.**
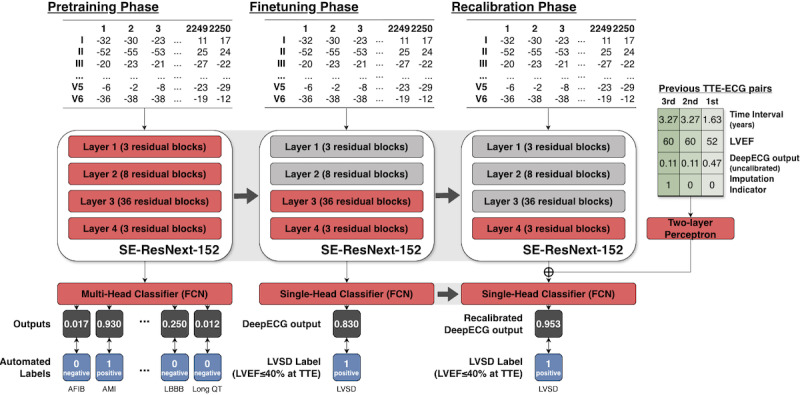
Schematic overview of the training process for artificial intelligence–augmented electrocardiogram SE-ResNeXt-152 was used as the backbone of the artificial intelligence–augmented electrocardiogram during the entire training process. AFIB: atrial fibrillation; AI-ECG: artificial intelligence–augmented electrocardiogram; AMI: acute myocardial infarction; ECG: electrocardiogram; FCN: fully connected network; LBBB: left bundle branch block; LVEF: left ventricular ejection fraction; LVSD: left ventricular systolic dysfunction; SE-ResNeXt: squeeze-and-excitation ResNeXt; TTE: transthoracic echocardiography; TTE-ECG: transthoracic echocardiography-electrocardiogram.

The development of the AI-ECG algorithm was structured into 3 sequential learning phases (Figure 1). In addition to the general framework of supervised learning, we introduced an initial pretraining stage by leveraging large-scale and automatically labeled ECG data. This step guided the model to capture broad, but clinically relevant, low-level ECG features that may not be apparent in smaller outcome-specific datasets, thus enabling more effective supervised training in the subsequent phase. The pretrained model parameters were then used to initialize the model for the second LVSD-specific fine-tuning phase.

Following fine-tuning, we implemented the third and final recalibration phase. Patients may exhibit different underlying cardiac conditions, including arrhythmias and prior infarction, resulting in variable correlations between ECG signals and true LVEF at the individual level. Therefore, the model was trained to adjust its predictions using each patient’s historical LVEF values and prior model outputs. This approach was designed to account for individual variability and temporal ECG trends relevant in real-world follow-up settings.

### First Pretraining Phase: Pretraining AI-ECG With Various ECG Features

The initial phase focused on pretraining the AI-ECG model using a large-scale ECG dataset from Hospital A, in which the labels were derived automatically. Specifically, we processed the analysis results from the MUSE system, including both free-text diagnostic statements and ECG numerical values (eg, PR interval, QRS duration, and QT interval).

The diagnostic strings were systematically parsed to identify and assign binary labels based on the presence of key clinical conditions, including arrhythmia (eg, AFIB, atrial flutter, premature atrial complex, and premature ventricular complex), myocardial infarction, and conduction abnormalities (eg, left bundle branch block [LBBB] and right bundle branch block). ECG values were thresholded to generate binary labels, including a prolonged PR interval (longer than 200 ms), a wide QRS complex (QRS duration >140 ms), and a prolonged QT interval (>450 ms in men and >470 ms in women after heart rate correction). This process resulted in 17 binary labels related to arrhythmias, 8 for conduction abnormalities, 8 for ischemic patterns, 7 for miscellaneous disease patterns, and 6 for binary labels derived from the thresholded numerical values. Each ECG was annotated with 46 binary labels to capture a comprehensive range of clinically relevant ECG features.

These binary labels were used as training targets for the initial pretraining phase of the AI-ECG model. Patients without eligible TTE-ECG pairs were included in the training phase. The model was trained to predict the presence of each binary label using the ECG signals of the training set. The cross-entropy loss on the validation split was monitored to determine the point of early termination of this pretraining phase.

### Second Fine-Tuning Phase: Fine-Tuning AI-ECG for LVSD Detection

In the second phase, the AI-ECG model was fine-tuned to detect LVSD using ECG records from the baseline TTE-ECG pair set at Hospital A in a supervised learning framework. The model weights were initialized during the pretraining phase. The first 2 convolutional blocks were kept frozen (ie, their weights were not updated during training) to ensure stable low-level feature extraction during the pretraining phase.

In contrast to the fine-tuned AI-ECG model (referred to as DeepECG LVSD) initialized from pretrained weights, a separate AI-ECG model was developed without pretraining under identical conditions. For this model, the weights were randomly initialized, and no layers were frozen during training. This nonpretrained AI-ECG model served as a control to assess the efficacy of the initial pretraining phase.

### Third Recalibration Phase: Recalibration Based on Previous LVEF and AI-ECG Outputs

Finally, we introduced a recalibration method to refine the model predictions by integrating previous LVEF measurements and prior model outputs. For recalibration, the model was supplemented with up to 3 previous TTE-ECG pairs for each patient as additional inputs. For each previous pair, we included (1) the actual LVEF value measured on TTE, (2) the uncalibrated DeepECG LVSD model output of the corresponding ECG, (3) the time interval between the previous ECG and the current ECG, and (4) an additional binary indicator representing missing data.

The calibrated model outputs of the previous ECGs were calculated in advance from the DeepECG LVSD models trained in the second fine-tuning phase. If a patient had fewer than 3 previous TTE-ECG pairs, missing data were imputed using a backward-filling strategy (ie, using the closest available prior values), and the binary indicator was marked as 1 for imputed (missing) values or 0 for observed (true) values. The resulting 12 features (4 features for the 3 previous pairs each) were used as the input for a 2-layer perceptron whose output was combined with the main model’s feature outputs using a point-wise sum operation before the final classification head. A second model with a different recalibration scheme—the recalibration masked to the LVEF—was trained. The LVEF measurements were excluded as input values, resulting in 9 features as 2-layer perceptron inputs. This model relies only on historical ECG records and does not require actual LVEF measurements from TTE for model inference.

Training for this recalibration phase was conducted using the follow-up TTE-ECG dataset from Hospital A. The weights were initialized in the second fine-tuning phase to preserve the learned ECG representations. The first 3 convolutional blocks were frozen during this phase to maintain the functionality trained during the first 2 stages.

### Model Architecture and Data Preprocessing

The backbone network of SE-ResNeXt152 was adopted as the model architecture and was composed of 152 1D convolutional layers nested in 4 convolutional blocks with squeeze and excitation modules [16,17]. This model architecture has been widely used in AI-ECG research and competitions, exhibiting competitive performance across various tasks. The final classification layer was modified during the learning phases as follows: (1) a multiheaded layer for the first pretraining phase, (2) a single-head layer for the second fine-tuning phase, and (3) a single-head layer connected to a 2-layer perceptron with a 12-dimensional vector input for the third recalibration phase. During the training phase, 9-second segments of ECGs were randomly cropped from the standard 10-second ECG recording as model input data. For evaluation, we cropped 10-second ECGs into 0‐9, 0.5‐9.5, and 1‐10 segments, and then averaged the output of each segment. Random cropping during training helped the AI-ECG model become location-invariant, and multiple cropping effectively acted as a model ensemble during evaluation. Subsequently, the cropped ECGs were downsampled or upsampled to 250 Hz to ensure a fixed input length. Finally, we performed bandpass filtering with a frequency band of 0.67 Hz to 40 Hz to remove baseline wandering and high-frequency noise.

### Training With Different Lead Configurations

To evaluate the possibility of broader application, separate models were trained using different numbers of ECG leads. The 12-lead, 6-lead (limb lead), and 1-lead (lead I) configurations were used. The models for each configuration were trained and validated independently following the same protocol. The first input layer of each model was modified to accept signals with channel counts of 12, 6, and 1. The primary analyses and main results of this study were based on the 12-lead configuration, which is consistent with the standard ECG acquisition.

### Performance Evaluation and Statistical Analysis

The model performance for detecting LVSD was evaluated using both internal (Hospital A) and external (Hospital B) test sets across all lead configurations (12-, 6-, and 1-lead). Following the second phase of fine-tuning, the DeepECG LVSD and AI-ECG models (without pretraining, as a control) were validated using baseline TTE-ECG pairs. To determine the effect of pretraining, area under the receiver operating curve (AUROC) values of the 2 models were compared. Subgroup analyses were performed using key clinical variables (demographics, *ICD-10* codes, and ECG characteristics) to assess the robustness across various subpopulations. These subgroup analyses primarily focused on the DeepECG LVSD model with a 12-lead configuration in an external validation set.

After the final recalibration phase, the recalibrated DeepECG LVSD model was evaluated for follow-up TTE-ECG pairs using ECGs, LVSD labels, and previous inference results. Additional validation was conducted on the second recalibrated DeepECG LVSD model with the LVEF values masked during training. The DeepECG LVSD model before recalibration was validated for direct comparison. AUROC comparisons of DeepECG LVSD models before and after recalibration were performed in the entire test cohort as well as within each subgroup to quantify performance improvements attributable to recalibration and identify subpopulations that benefit from the recalibration process.

The raw model output was a continuous probability value between 0 and 1, which represented the estimated likelihood of LVSD. The AUROC was calculated to quantify discriminative performance. For binary classification, the threshold was determined using Hospital A’s validation set, which yielded the highest Youden index [18] (sensitivity + specificity – 1). The threshold was applied to calculate sensitivity, specificity, accuracy, positive predictive value (PPV), and negative predictive value (NPV). For each metric, 95% CIs were estimated using the DeLong method [19] for the AUROC and the Clopper-Pearson exact method [20] for the sensitivity, specificity, PPV, and NPV. For subgroup analysis, all 95% CIs were estimated by bootstrapping over 1000 iterations. All statistical AUROC comparisons were conducted using the DeLong method, and the corresponding *P* values were reported.

Baseline characteristics are summarized as mean (SD) for continuous variables and as frequency (%) for binary variables. Group comparisons between the training, validation, and test sets of Hospital A, and between the test sets of Hospitals A and B were performed. Continuous variables were tested on ANOVA or Student *t* test and categorical variables were tested using the chi-square test. Analysis of baseline characteristics was performed on the baseline TTE-ECG pairs to ensure nonduplicated, patient-level sampling.

### Ethical Considerations

This study was approved by the Ethics Committees of Hallym Sacred Heart Hospital (IRB No. 2022-03-016-008) and Wonju Severance Christian Hospital (IRB No. CR322014). Due to the retrospective nature of the study, the requirement for informed consent was waived by both institutional review boards. All data used in this study were fully deidentified prior to analysis, and no personally identifiable information, including patient names, hospital numbers, or dates of birth, was accessed by the research team, ensuring the privacy and confidentiality of the participants. No compensation was provided to participants, as this study exclusively involved the secondary analysis of preexisting, de-identified clinical data.

## Results

### Baseline Characteristics of Study Population

**Figure 2.**
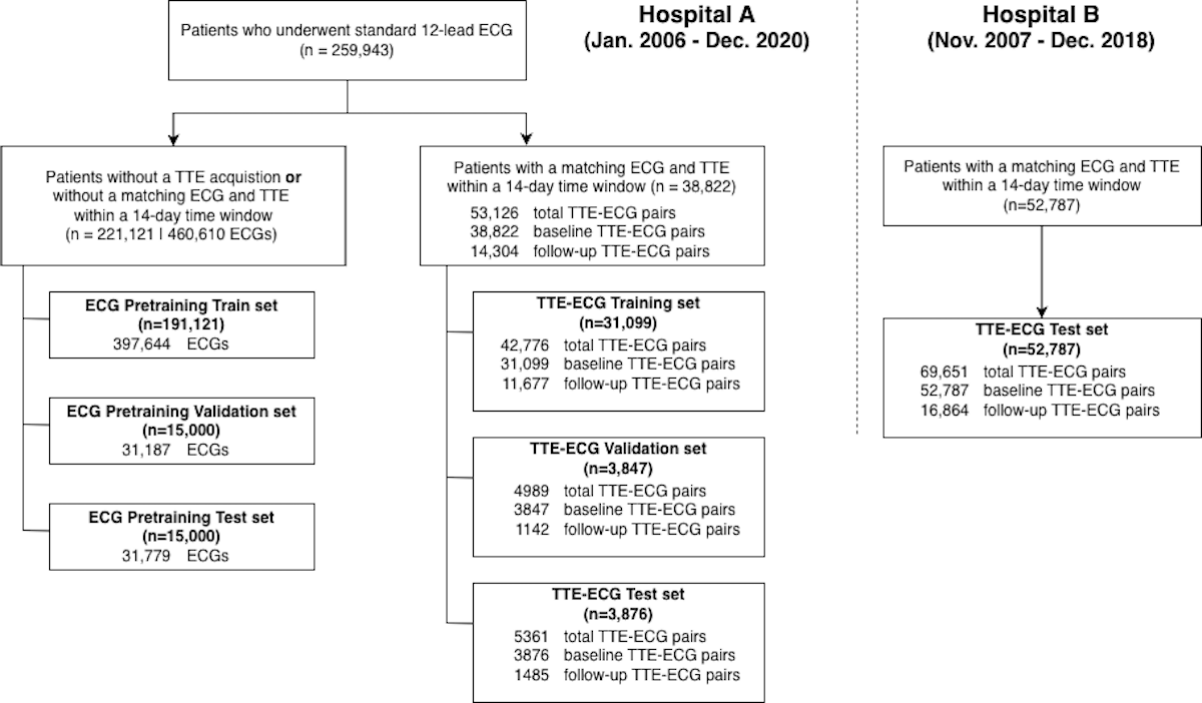
Study flowchart summarizing the data collection and preprocessing steps performed in this study. ECG: electrocardiogram; TTE: transthoracic echocardiography; TTE-ECG: transthoracic echocardiography-electrocardiogram.

In total, 31,099 training participants, 3847 validation participants, and 3876 test participants from Hospital A, and 52,787 external test participants from Hospital B contributed to one or more TTE-ECG pairs in this study (Figure 2). The clinical, ECG, and echocardiographic parameters for each dataset are summarized in Table 1. In the Hospital A test set, the mean age was 63.3 (15.5) years, 51.9% (2012/3876) were men, and the mean LVEF was 60% (11.6%). In the Hospital B test set, the mean age was 63.7 (14.4) years, 53.8% (28,391/52,787) were men, and the mean LVEF was 62.7% (10.2%). No significant differences in demographics, TTE, and ECG measurements were observed between the training, validation, and test sets at Hospital A. However, the test set of Hospital B exhibited a higher ratio of men, a higher mean LVEF, and a higher proportion of AFIB or atrial flutter in the ECG than the test set of Hospital A.

**Table 1.** Baseline characteristics of the patients for baseline TTE-ECG^a^ pairs

Characteristics	Hospital A				Hospital B (n=52,787)	
	Train (n=31,099)	Validation (n=3847)	Test (n=3876)	*P* value^b^	*P* value^c^
Demographic information						
Age (years), mean (SD)	63.4 (15.1)	63.4 (15.0)	63.3 (15.5)	.98	63.7 (14.4)	.19
Men, n (%)	16,339 (52.5)	2014 (52.4)	2012 (51.9)	.75	28,391 (53.8)	.02
BMI (kg/m^2^), mean (SD)	24.3 (3.8)	24.3 (3.9)	24.2 (3.7)	.34	24.3 (3.7)	.03
DM^d^, n (%)	7034 (22.6)	887 (23.1)	878 (22.7)	.83	11,795 (22.3)	.67
HTN^e^, n (%)	16,237 (52.2)	1953 (50.8)	2003 (51.7)	.22	23,050 (43.7)	<.001
IHD^f^, n (%)	1904 (6.1)	257 (6.7)	238 (6.1)	.40	3098 (5.9)	.51
CKD^g^, n (%)	1989 (6.4)	253 (6.6)	263 (6.8)	.61	3280 (6.2)	.17
LVSD^h^, n (%)	2419 (7.8)	300 (7.8)	310 (8.0)	.89	2563 (4.9)	<.001
Echocardiographic or electrocardiographic information						
LVEF^i^ (%), mean (SD)	60.2 (11.5)	60.0 (11.5)	60.0 (11.6)	.51	62.7 (10.2)	<.001
E/e**’**, mean (SD)	9.6 (4.5)	9.7 (4.7)	9.7 (4.7)	.35	12.9 (5.2)	<.001
HR^j^ (bpm), mean (SD)	74.6 (16.7)	74.1 (16.3)	74.8 (17.1)	.12	75.5 (18.1)	.01
PR (ms), mean (SD)	165.3 (26.2)	165.8 (27.0)	165.2 (25.6)	.55	164.0 (27.1)	.007
QRS (ms), mean (SD)	94.1 (16.0)	94.4 (16.0)	94.2 (15.8)	.70	92.9 (16.4)	<.001
QTc (ms), mean (SD)	436.5 (33.1)	436.5 (33.6)	436.5 (34.1)	.99	441.6 (37.4)	<.001
AFIB^k^/AFL^l^, n (%)	2297 (7.4)	278 (7.2)	273 (7)	.72	4963 (9.4)	<.001

a TTE-ECG: transthoracic echocardiography–electrocardiogram.

b *P* values for group comparisons among the train, validation, and test sets of Hospital A were calculated using ANOVA for continuous variables and the chi-square test for categorical variables.

c *P* values for group comparisons between the test set of Hospital A and Hospital B were calculated using the Student *t* test for continuous variables and chi-square test for categorical variables.

d DM: diabetes mellitus.

e HTN: hypertension.

f IHD: ischemic heart disease.

g CKD: chronic kidney disease.

h LVSD: left ventricular systolic dysfunction.

i LVEF: left ventricular ejection fraction.

j HR: heart rate.

k AFIB: atrial fibrillation.

l AFL: atrial flutter.

### LVSD Detection in Baseline TTE-ECG Pairs

After the second fine-tuning phase, the DeepECG LVSD and AI-ECG models (trained without pretraining) were validated against the LVSD label of the baseline TTE-ECG pairs (first pair for each patient) of hospitals A and B. The standard 12-lead DeepECG LVSD model exhibited an AUROC of 0.953 (95% CI 0.941‐0.965) in internal validation and 0.947 (95% CI 0.943‐0.951) in external validation (Table 2). Using thresholds that maximized the Youden index, the binary classification performance showed a sensitivity of 0.903 (95% CI 0.865‐0.934) and a specificity of 0.844 (95% CI 0.832‐0.856) in the internal validation and a sensitivity of 0.915 (95% CI 0.904‐0.926) and a specificity of 0.847 (95% CI 0.844‐0.850) in the external validation.

**Table 2.** LVSD^a^ detection performance of the AI-ECG^b^ model and DeepECG LVSD model, evaluated within baseline TTE-ECG^c^ pairs.

Performance		12-lead, value (95% CI)	6-lead, value (95% CI)	1-lead, value (95% CI)
Internal validation (Hospital A)				
AI-ECG without pretraining	AUROC^d^	0.939 (0.926‐0.953)^e^	0.927 (0.912‐0.942)	0.919 (0.905‐0.934)
	Sensitivity	0.897 (0.857‐0.928)	0.858 (0.814‐0.895)	0.910 (0.872‐0.939)
	Specificity	0.834 (0.822‐0.846)	0.840 (0.828‐0.852)	0.777 (0.764‐0.791)
	PPV^f^	0.320 (0.289‐0.352)	0.318 (0.287‐0.351)	0.262 (0.236‐0.289)
	NPV^g^	0.989 (0.985‐0.992)	0.986 (0.981‐0.989)	0.990 (0.986‐0.993)
DeepECG LVSD	AUROC	0.953 (0.941‐0.965)	0.943 (0.930‐0.955)	0.930 (0.916‐0.944)
	Sensitivity	0.903 (0.865‐0.934)	0.935 (0.902‐0.960)	0.913 (0.876‐0.942)
	Specificity	0.844 (0.832‐0.856)	0.789 (0.776‐0.802)	0.787 (0.773‐0.800)
	PPV	0.335 (0.303‐0.368)	0.278 (0.251‐0.307)	0.271 (0.244‐0.299)
	NPV	0.990 (0.986‐0.993)	0.993 (0.990‐0.995)	0.990 (0.987‐0.993)
External validation (Hospital B)				
AI-ECG without pretraining	AUROC	0.937 (0.933‐0.941)	0.926 (0.921‐0.930)	0.916 (0.911‐0.921)
	Sensitivity	0.890 (0.878‐0.902)	0.895 (0.883‐0.907)	0.854 (0.839‐0.867)
	Specificity	0.844 (0.841‐0.847)	0.814 (0.811‐0.817)	0.840 (0.837‐0.843)
	PPV	0.226 (0.217‐0.234)	0.197 (0.190‐0.205)	0.214 (0.206‐0.222)
	NPV	0.993 (0.993‐0.994)	0.993 (0.993‐0.994)	0.991 (0.990‐0.992)
DeepECG LVSD	AUROC	0.947 (0.943‐0.951)	0.936 (0.932‐0.940)	0.922 (0.918‐0.927)
	Sensitivity	0.915 (0.904‐0.926)	0.909 (0.898‐0.920)	0.877 (0.863‐0.889)
	Specificity	0.847 (0.844‐0.850)	0.831 (0.828‐0.835)	0.823 (0.820‐0.826)
	PPV	0.234 (0.226‐0.243)	0.216 (0.208‐0.224)	0.202 (0.194‐0.209)
	NPV	0.995 (0.994‐0.996)	0.994 (0.994‐0.995)	0.992 (0.992‐0.993)

a LVSD: left ventricular systolic dysfunction.

b AI-ECG: artificial intelligence–augmented electrocardiogram.

c TTE-ECG: transthoracic echocardiography-electrocardiogram.

d AUROC: area under the receiver operating curve.

e CIs (95%) are estimated from the DeLong method for AUROC and the Clopper-Pearson exact method for sensitivity, specificity, positive predictive value, and negative predictive value.

f PPV: positive predictive value.

g NPV: negative predictive value.

The models using 6-lead and 1-lead ECGs also showed adequate performance with internal AUROC 0.943 (95% CI 0.930‐0.955) and external AUROC 0.936 (95% CI 0.932‐0.940) for 6-lead models, and internal AUROC 0.930 (95% CI 0.916‐0.944) and external AUROC 0.922 (95% CI 0.918‐0.927) for 1-lead models.

**Figure 3.**
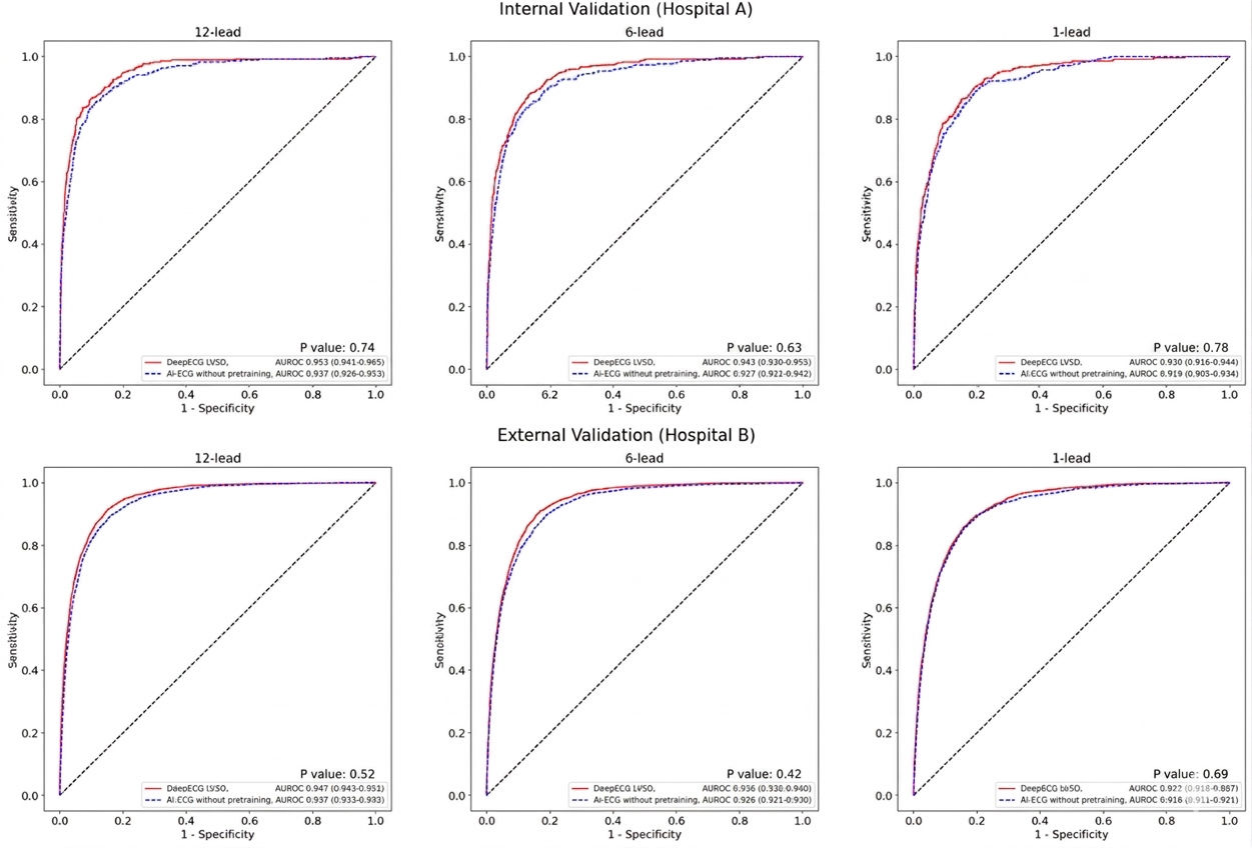
Receiver operating characteristic curve for left ventricular systolic dysfunction detection performance of the artificial intelligence–augmented electrocardiogram model and the DeepECG LVSD model, evaluated within baseline transthoracic echocardiography-electrocardiogram pairs. AI-ECG: artificial intelligence–augmented electrocardiogram; AUROC: area under the receiver operating characteristic; ECG: electrocardiogram; LVSD: left ventricular systolic dysfunction.

Notably, when compared with the AI-ECG models without pretraining, the pretrained DeepECG LVSD models demonstrated a higher performance, though not statistically significant (Figure 3). The 12-lead AI-ECG models without pretraining showed an AUROC of 0.939 (95% CI 0.926‐0.953) for the internal validation set and 0.937 (95% CI 0.933‐0.941) for the external validation set.

Subgroup analysis revealed that the 12-lead DeepECG LVSD model maintained robust discriminative performance across most clinical variables (Table 3). A notable decline in performance was observed in some subgroups. In patients with AFIB or flutter, the AUROC decreased to 0.875 (95% CI 0.858‐0.890), accompanied by a reduction in specificity to 0.578 (95% CI 0.564‐0.592) and PPV to 0.174 (95% CI 0.158‐0.190). LBBB was also associated with a low AUROC of 0.859 (95% CI 0.828‐0.890) and a lower specificity of 0.219 (95% CI 0.177‐0.266). Other conditions associated with a specificity below 0.700 included the presence of ischemic heart disease, a wide QRS complex, and a prolonged QT interval.

**Table 3.** Subgroup analysis on LVSD^a^ detection performance of the DeepECG LVSD model, evaluated within 12-lead, external, baseline TTE-ECG^b^ pairs.^c^

Subgroup		DeepECG LVSD				
	n/N (%)^d^	AUROC^e^ (95% CI)	Sensitivity (95% CI)	Specificity (95% CI)	PPV^f^ (95% CI)	NPV^g^ (95% CI)
Age ≤70	1268/33,176 (3.8%)	0.957 (0.952‐0.961)	0.914 (0.899‐0.929)	0.874 (0.870‐0.878)	0.224 (0.213‐0.236)	0.996 (0.995‐0.997)
Age >70	1295/19,611 (6.6%)	0.930 (0.924‐0.936)	0.918 (0.903‐0.932)	0.797 (0.791‐0.802)	0.242 (0.231‐0.254)	0.993 (0.991‐0.994)
Male	1550/28,391 (5.5%)	0.945 (0.940‐0.950)	0.920 (0.906‐0.933)	0.831 (0.826‐0.835)	0.239 (0.228‐0.250)	0.994 (0.993‐0.995)
Female	1011/24,338 (4.2%)	0.949 (0.944‐0.954)	0.910 (0.893‐0.927)	0.864 (0.859‐0.868)	0.224 (0.212‐0.236)	0.996 (0.995‐0.996)
DM^h^ (+)	836/11,795 (7.1%)	0.939 (0.932‐0.946)	0.926 (0.906‐0.943)	0.809 (0.803‐0.816)	0.270 (0.255‐0.286)	0.993 (0.991‐0.995)
DM (–)	1727/40,992 (4.2%)	0.949 (0.944‐0.953)	0.911 (0.898‐0.925)	0.856 (0.853‐0.860)	0.218 (0.209‐0.227)	0.995 (0.995‐0.996)
HTN^i^ (+)	1188/23,050 (5.2%)	0.948 (0.943‐0.952)	0.936 (0.921‐0.949)	0.832 (0.827‐0.837)	0.233 (0.220‐0.245)	0.996 (0.995‐0.997)
HTN (–)	1375/29,737 (4.6%)	0.946 (0.941‐0.951)	0.899 (0.882‐0.914)	0.856 (0.852‐0.861)	0.233 (0.222‐0.245)	0.994 (0.993‐0.995)
IHD^j^ (+)	497/3098 (16.0%)	0.868 (0.852‐0.883)	0.934 (0.910‐0.955)	0.596 (0.577‐0.614)	0.306 (0.283‐0.330)	0.979 (0.972‐0.986)
IHD (–)	2066/49,689 (4.2%)	0.950 (0.947‐0.954)	0.912 (0.900‐0.925)	0.860 (0.857‐0.863)	0.220 (0.211‐0.229)	0.996 (0.995‐0.996)
CKD^k^ (+)	326/3280 (9.9%)	0.906 (0.890‐0.920)	0.926 (0.898‐0.952)	0.706 (0.690‐0.724)	0.258 (0.233‐0.282)	0.989 (0.984‐0.993)
CKD (–)	2237/49,507 (4.5%)	0.950 (0.946‐0.953)	0.915 (0.903‐0.925)	0.855 (0.852‐0.858)	0.230 (0.220‐0.238)	0.995 (0.995‐0.996)
Sinus rhythm	2014/46,763 (4.3%)	0.956 (0.952‐0.959)	0.910 (0.897‐0.922)	0.877 (0.874‐0.880)	0.250 (0.241‐0.260)	0.995 (0.995‐0.996)
AFIB^l^/AFL^m^	428/4963 (8.6%)	0.875 (0.858‐0.890)	0.944 (0.920‐0.965)	0.578 (0.564‐0.592)	0.174 (0.158‐0.190)	0.991 (0.987‐0.994)
LBBB^n^	220/553 (39.8%)	0.859 (0.828‐0.890)	0.991 (0.977‐1.000)	0.219 (0.177‐0.266)	0.456 (0.412‐0.501)	0.973 (0.933‐1.000)
RBBB^o^	199/3420 (5.8%)	0.927 (0.910‐0.941)	0.910 (0.867‐0.947)	0.803 (0.788‐0.816)	0.222 (0.194‐0.249)	0.993 (0.990‐0.996)
PR >200 ms	156/3696 (4.2%)	0.935 (0.913‐0.956)	0.878 (0.821‐0.927)	0.870 (0.858‐0.881)	0.229 (0.195‐0.262)	0.994 (0.991‐0.996)
QRS >140 ms	238/1317 (18.1%)	0.927 (0.910‐0.942)	0.979 (0.961‐0.996)	0.653 (0.623‐0.682)	0.384 (0.348‐0.424)	0.993 (0.987‐0.999)
Prolonged QTc^p^^,^^q^	1566/13,518 (11.6%)	0.909 (0.902‐0.916)	0.939 (0.927‐0.952)	0.671 (0.663‐0.680)	0.272 (0.260‐0.285)	0.988 (0.986‐0.991)

a LVSD: left ventricular systolic dysfunction.

b TTE-ECG: transthoracic echocardiography-electrocardiogram.

c All CIs (95%) are estimated from bootstrapping 1000 iterations.

d Values are presented as number of cases/total number of subjects (%).

e AUROC: area under the receiver operating curve.

f PPV: positive predictive value.

g NPV: negative predictive value.

h DM: diabetes mellitus.

i HTN: hypertension.

j IHD: ischemic heart disease.

k CKD: chronic kidney disease.

l AFIB: atrial fibrillation.

m AFL: atrial flutter.

n LBBB: left bundle branch block.

o RBBB: right bundle branch block.

p Prolonged QTc is defined as > 450 ms for men and > 470 ms for women after heart rate correction.

q QTc: heart-rate-corrected QT interval.

### LVSD Detection in Follow-Up TTE-ECG Pairs

**Figure 4.**
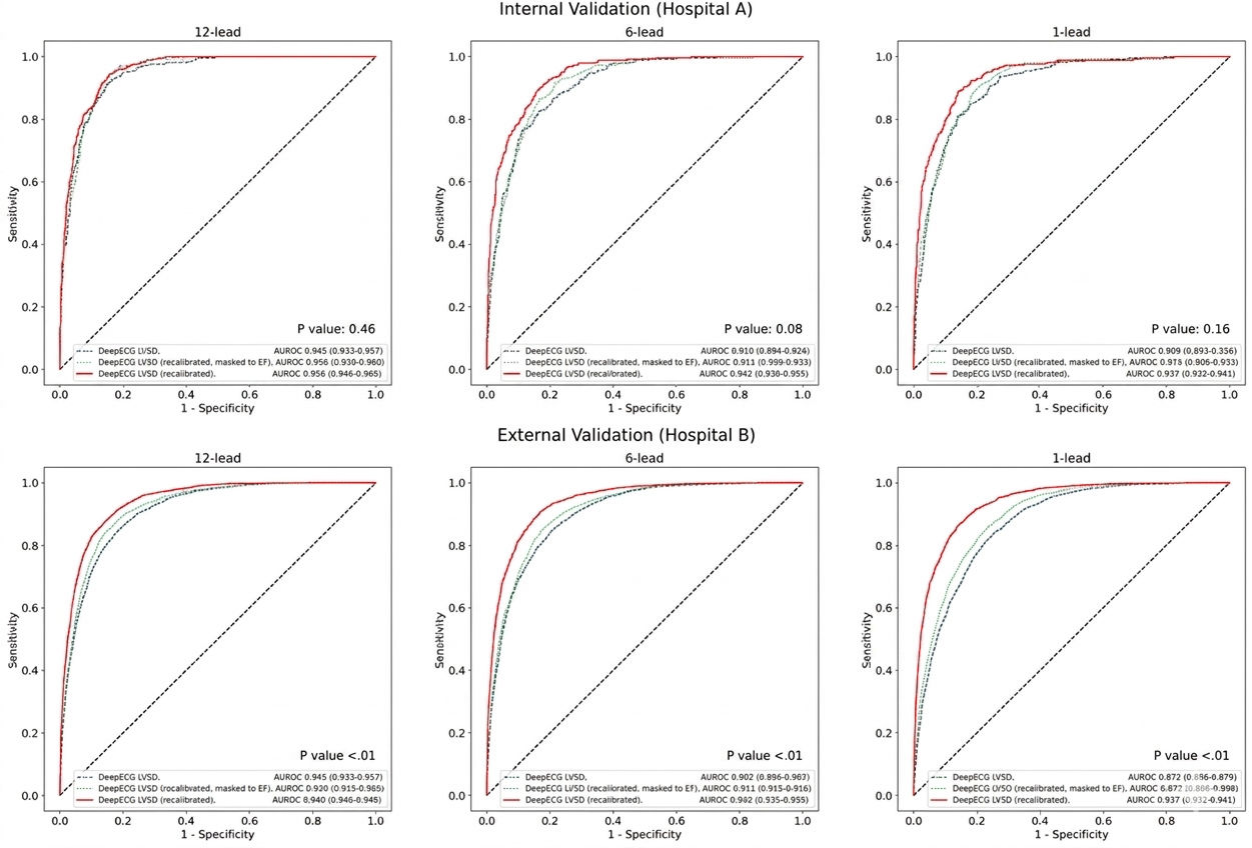
Receiver operating characteristic curve for left ventricular systolic dysfunction detection performance of the DeepECG LVSD model, before and after recalibration, evaluated within the follow-up transthoracic echocardiography-electrocardiogram pairs. AUROC: area under the receiver operating characteristic; ECG: electrocardiogram; LVEF: left ventricular ejection fraction; LVSD: left ventricular systolic dysfunction.

The recalibrated DeepECG LVSD models demonstrated high performance when validated on ECGs and LVSD labels from follow-up TTE-ECG pairs (ie, nonfirst pairs) in both hospitals A and B (Table 4**)**. The 12-lead model achieved an AUROC of 0.956 (95% CI 0.946‐0.965) for internal validation and 0.940 (95% CI 0.936‐0.945) for external validation, representing a modest improvement over the uncalibrated DeepECG LVSD model with an internal AUROC of 0.945 (95% CI 0.933‐0.957) and an external AUROC of 0.910 (95% CI 0.905‐0.916). Model recalibration without LVEF values showed a subtle performance gain over the uncalibrated model, yielding an internal AUROC of 0.950 (95% CI 0.939‐0.960) and an external AUROC of 0.920 (95% CI 0.915‐0.925). AUROC comparisons showed statistically significant differences between the recalibrated and uncalibrated DeepECG LVSD models among the external test sets (Figure 4). Also, at clinically relevant operating points, recalibrated models outperformed their uncalibrated counterparts. Specifically, when sensitivity was fixed at 90%, recalibration yielded higher specificity. Likewise, when specificity was fixed at 90%, recalibrated models achieved higher sensitivity (Table S1 and S2 in Multimedia Appendix 1).

**Table 4.** LVSD^a^ detection performance of the DeepECG LVSD model before and after recalibration, evaluated within the follow-up TTE-ECG^b^ pairs of internal and external validation.

Performance		12-lead, value (95% CI)	6-lead, value (95% CI)	1-lead, value (95% CI)
Internal validation (Hospital A)				
DeepECG LVSD	AUROC^c^	0.945 (0.933‐0.957)	0.910 (0.894‐0.926)	0.909 (0.893‐0.926)
	Sensitivity	0.914 (0.875‐0.943)	0.848 (0.802‐0.888)	0.862 (0.817‐0.900)
	Specificity	0.852 (0.831‐0.871)	0.801 (0.778‐0.822)	0.799 (0.776‐0.821)
	PPV^d^	0.600 (0.552‐0.646)	0.508 (0.463‐0.554)	0.510 (0.465‐0.555)
	NPV^e^	0.976 (0.966‐0.984)	0.956 (0.943‐0.967)	0.960 (0.947‐0.970)
DeepECG LVSD (recalibrated, masked to LVEF^f^)	AUROC	0.950 (0.939‐0.960)	0.918 (0.903‐0.933)	0.918 (0.902‐0.933)
	Sensitivity	0.903 (0.863‐0.935)	0.893 (0.852‐0.926)	0.928 (0.891‐0.955)
	Specificity	0.855 (0.835‐0.874)	0.794 (0.771‐0.816)	0.756 (0.732‐0.780)
	PPV	0.602 (0.555‐0.649)	0.513 (0.468‐0.557)	0.480 (0.438‐0.523)
	NPV	0.973 (0.963‐0.981)	0.968 (0.957‐0.978)	0.977 (0.967‐0.985)
DeepECG LVSD (recalibrated)	AUROC	0.956 (0.946‐0.965)	0.942 (0.930‐0.955)	0.939 (0.925‐0.953)
	Sensitivity	0.828 (0.779‐0.869)	0.869 (0.825‐0.906)	0.924 (0.887‐0.952)
	Specificity	0.912 (0.896‐0.927)	0.854 (0.833‐0.872)	0.814 (0.792‐0.835)
	PPV	0.696 (0.644‐0.744)	0.590 (0.542‐0.637)	0.547 (0.502‐0.592)
	NPV	0.956 (0.944‐0.967)	0.964 (0.952‐0.974)	0.978 (0.968‐0.985)
External validation (Hospital B)				
DeepECG LVSD	AUROC	0.910 (0.905‐0.916)	0.902 (0.896‐0.907)	0.872 (0.866‐0.879)
	Sensitivity	0.894 (0.880‐0.906)	0.913 (0.901‐0.924)	0.901 (0.888‐0.913)
	Specificity	0.763 (0.757‐0.770)	0.705 (0.697‐0.712)	0.670 (0.662‐0.677)
	PPV	0.378 (0.366‐0.391)	0.332 (0.321‐0.344)	0.305 (0.294‐0.316)
	NPV	0.978 (0.975‐0.981)	0.980 (0.978‐0.983)	0.977 (0.974‐0.979)
DeepECG LVSD (recalibrated, masked to LVEF)	AUROC	0.920 (0.915‐0.925)	0.911 (0.905‐0.916)	0.892 (0.886‐0.898)
	Sensitivity	0.888 (0.875‐0.901)	0.856 (0.842‐0.870)	0.861 (0.846‐0.874)
	Specificity	0.810 (0.804‐0.817)	0.819 (0.812‐0.825)	0.763 (0.756‐0.770)
	PPV	0.430 (0.416‐0.444)	0.432 (0.418‐0.446)	0.369 (0.357‐0.382)
	NPV	0.978 (0.976‐0.981)	0.973 (0.970‐0.975)	0.971 (0.968‐0.974)
DeepECG LVSD (recalibrated)	AUROC	0.940 (0.936‐0.945)	0.939 (0.935‐0.944)	0.937 (0.932‐0.941)
	Sensitivity	0.874 (0.860‐0.887)	0.905 (0.892‐0.916)	0.864 (0.849‐0.877)
	Specificity	0.859 (0.854‐0.865)	0.833 (0.827‐0.839)	0.859 (0.853‐0.865)
	PPV	0.500 (0.485‐0.516)	0.466 (0.452‐0.481)	0.497 (0.481‐0.512)
	NPV	0.977 (0.974‐0.979)	0.982 (0.979‐0.984)	0.975 (0.972‐0.978)

a LVSD: left ventricular systolic dysfunction.

b TTE-ECG: transthoracic echocardiography-electrocardiogram.

c AUROC: area under the receiver operating curve.

d PPV: positive predictive value.

e NPV: negative predictive value.

f LVEF: left ventricular ejection fraction.

Subgroup analysis revealed that all subpopulations consistently showed statistically significant differences between the AUROC of the recalibrated and uncalibrated standard 12-lead DeepECG LVSD models (Table 5). In these subgroups, the recalibrated models also demonstrated substantially higher specificity compared with the uncalibrated model when evaluated at each model’s respective decision thresholds.

**Table 5.** Subgroup analysis and AUROC^a^ comparisons for LVSD^b^ detection performance of DeepECG LVSD before and after recalibration, evaluated within 12-lead, external, follow-up TTE-ECG^c^ pairs.^d^

Subgroup		DeepECG LVSD (recalibrated)			DeepECG LVSD			
	n/N (%)^e^	AUROC (95% CI)	Sensitivity (95% CI)	Specificity (95% CI)	AUROC (95% CI)	Sensitivity (95% CI)	Specificity (95% CI)	*P* value^f^
Age ≤70	1245/10,002(12.5%)	0.947 (0.941‐0.952)	0.802 (0.780‐0.825)	0.920 (0.914‐0.925)	0.921 (0.915‐0.928)	0.928 (0.913‐0.943)	0.733 (0.724‐0.742)	.001
Age >70	1095/6862(16.0%)	0.931 (0.924‐0.938)	0.767 (0.741‐0.793)	0.914 (0.907‐0.921)	0.894 (0.884‐0.903)	0.923 (0.908‐0.940)	0.666 (0.654‐0.677)	<.001
Male	1404/9259(15.2%)	0.941 (0.936‐0.947)	0.818 (0.797‐0.839)	0.905 (0.898‐0.910)	0.912 (0.905‐0.919)	0.928 (0.914‐0.942)	0.706 (0.696‐0.716)	<.001
Female	933/7598(12.3%)	0.938 (0.931‐0.945)	0.740 (0.712‐0.766)	0.933 (0.926‐0.938)	0.908 (0.900‐0.915)	0.922 (0.905‐0.939)	0.707 (0.696‐0.718)	.002
DM^g^ (+)	908/5061(17.9%)	0.933 (0.926‐0.940)	0.779 (0.753‐0.804)	0.902 (0.893‐0.911)	0.903 (0.892‐0.912)	0.941 (0.924‐0.956)	0.664 (0.650‐0.679)	<.001
DM (–)	1432/11,803(12.1%)	0.943 (0.937‐0.948)	0.791 (0.769‐0.811)	0.923 (0.918‐0.928)	0.912 (0.905‐0.919)	0.916 (0.902‐0.931)	0.723 (0.714‐0.733)	<.001
HTN^h^ (+)	1248/8905(14.0%)	0.939 (0.933‐0.945)	0.796 (0.774‐0.816)	0.914 (0.907‐0.920)	0.908 (0.900‐0.915)	0.932 (0.918‐0.945)	0.692 (0.681‐0.702)	<.001
HTN (–)	1092/7959(13.7%)	0.942 (0.935‐0.948)	0.775 (0.748‐0.802)	0.922 (0.915‐0.928)	0.913 (0.905‐0.921)	0.918 (0.903‐0.934)	0.723 (0.712‐0.733)	<.001
IHD^i^ (+)	552/2147(25.7%)	0.914 (0.902‐0.927)	0.853 (0.822‐0.884)	0.824 (0.806‐0.845)	0.876 (0.861‐0.890)	0.942 (0.922‐0.961)	0.562 (0.539‐0.586)	<.001
IHD (–)	1788/14,717(12.2%)	0.942 (0.937‐0.947)	0.765 (0.744‐0.785)	0.929 (0.924‐0.933)	0.913 (0.907‐0.919)	0.921 (0.908‐0.933)	0.724 (0.716‐0.731)	<.001
CKD^j^ (+)	400/1917(20.9%)	0.911 (0.898‐0.924)	0.790 (0.752‐0.828)	0.866 (0.849‐0.882)	0.872 (0.854‐0.890)	0.955 (0.933‐0.973)	0.545 (0.520‐0.570)	.005
CKD (–)	1940/14,947(13.0%)	0.943 (0.939‐0.948)	0.785 (0.766‐0.803)	0.923 (0.919‐0.928)	0.914 (0.908‐0.920)	0.920 (0.907‐0.932)	0.725 (0.717‐0.732)	<.001
Sinus rhythm	1704/13,148(13.0%)	0.946 (0.941‐0.950)	0.787 (0.768‐0.807)	0.923 (0.918‐0.928)	0.922 (0.916‐0.928)	0.920 (0.907‐0.932)	0.756 (0.748‐0.764)	<.001
AFIB^k^/AFL^l^	513/3168(16.2%)	0.926 (0.915‐0.937)	0.786 (0.750‐0.822)	0.900 (0.888‐0.911)	0.869 (0.854‐0.885)	0.936 (0.914‐0.956)	0.518 (0.500‐0.537)	.001
LBBB^m^	247/470(52.6%)	0.872 (0.841‐0.901)	0.947 (0.917‐0.973)	0.614 (0.554‐0.680)	0.819 (0.781‐0.855)	0.988 (0.972‐1.000)	0.184 (0.136‐0.238)	.03
RBBB^n^	205/1440(14.2%)	0.916 (0.900‐0.933)	0.785 (0.727‐0.839)	0.881 (0.863‐0.900)	0.853 (0.829‐0.877)	0.878 (0.835‐0.921)	0.669 (0.644‐0.696)	.01
PR > 200	219/1444(15.2%)	0.944 (0.930‐0.955)	0.813 (0.759‐0.863)	0.902 (0.885‐0.919)	0.908 (0.890‐0.924)	0.909 (0.867‐0.944)	0.749 (0.725‐0.773)	.04
QRS >140	285/770(37.0%)	0.916 (0.894‐0.934)	0.926 (0.893‐0.956)	0.755 (0.713‐0.791)	0.860 (0.830‐0.885)	0.954 (0.927‐0.978)	0.470 (0.427‐0.513)	.002
Prolonged QTc^o^^,^^p^	1512/6270(24.1%)	0.911 (0.904‐0.919)	0.814 (0.795‐0.833)	0.852 (0.841‐0.862)	0.869 (0.858‐0.878)	0.935 (0.922‐0.947)	0.538 (0.525‐0.552)	<.001

a AUROC: area under the receiver operating curve.

b LVSD: left ventricular systolic dysfunction.

c TTE-ECG: transthoracic echocardiography-electrocardiogram.

d All CIs (95%) are estimated from bootstrapping 1000 iterations.

e Values are presented as number of cases/total number of subjects (%).

f *P* value for the DeLong test between the recalibrated DeepECG LVSD model and the uncalibrated DeepECG LVSD model is calculated.

g DM: diabetes mellitus.

h HTN: hypertension.

i IHD: ischemic heart disease.

j CKD: chronic kidney disease.

k AFIB: atrial fibrillation.

l AFL: atrial flutter.

m LBBB: left bundle branch block.

n RBBB: right bundle branch block.

o Prolonged QTc is defined as >450 ms for men and >470 ms for women after heart rate correction.

p QTc: heart-rate-corrected QT interval.

## Discussion

### Principal Findings

In this multicenter retrospective analysis, we developed and validated DeepECG LVSD models capable of detecting LVSD from routine ECGs and achieved state-of-the-art performance across both internal and external cohorts. This resulted from a 3-stage training framework, including an initial pretraining phase using a large-scale, automatically labeled ECG dataset, and a final recalibration phase that adjusts model predictions based on prior LVEF measurements and previous AI-ECG outputs.

### Clinical Covariates in AI-ECG

Certain comorbidities, such as AFIB [6,10,14,15] or LBBB [10,15], can adversely affect the diagnostic accuracy of AI-ECG models. In line with prior findings, our analysis also showed declines in AUROC within these subgroups. However, the decline in specificity was considerably more pronounced than would be anticipated from AUROC alone. Specifically, patients without LVSD but with significant comorbidities frequently generated elevated model scores, increasing the false-positive rate. This pattern is consistent with the findings [10,15]. Harmon et al [10] have reported substantial decreases in AUROC and specificity in patients with AFIB (AUROC 0.826) or LBBB (AUROC 0.791) compared to the general population (AUROC 0.903), with specificity plummeting as low as 0.745 for AFIB and 0.364 for LBBB. Interestingly, while Harmon et al [10] recommended threshold adjustment, their suggestion was limited to patients with entirely normal ECGs rather than to broader subpopulations.

These performance drops can be explained by confounding variables. A well-known example in the AI literature is the effect of age as a confounder in models diagnosing HIV from magnetic resonance imaging [21]; age correlates with both imaging features and disease prevalence, thus influencing model predictions. Similarly, LBBB [22] and AFIB [23] are established risk factors for HF, and their correlation with LVSD is evident at the population level. As a result, models may exhibit a higher likelihood of positive classification among patients with such comorbidities, driven by population-level correlations. Under external evaluation or individual application, where the relationship between the confounder (eg, LBBB or AFIB) and target condition (LVSD) may not hold, the model may show decreased performance. Although our recalibration methods have empirically helped circumvent these effects, the direct means to mitigate confounding factors remain an open area of investigation in the AI-ECG literature.

Moreover, the AUROC primarily quantifies discrimination based on continuous risk scores and reflects rank-based information, which does not necessarily translate into clinical utility at predetermined decision thresholds. Thus, the subgroup AUROC alone may mask clinically significant degradations in binary performance metrics, such as specificity and sensitivity, which are directly relevant to clinical application. We suggest that future studies include comprehensive subgroup analyses that incorporate threshold-based performance metrics rather than stratified AUROC. Although current guidelines recommend subgroup analysis, the choice of performance metrics to report is ambiguous [24-26]; clearer reporting of clinically actionable, threshold-specific metrics could improve their relevance to real-world applications.

### Model Recalibration and Clinical Utility

To address these challenges, we introduced a patient-specific recalibration strategy that leverages prior LVEF values and historical DeepECG LVSD predictions. Rather than modifying model architecture or incorporating comorbidity information as direct inputs, recalibration adjusts patient-level risk estimates while preserving workflow simplicity. This strategy was designed to stabilize model behavior at a fixed decision threshold, not by altering the threshold itself. By recalibrating risk estimates at the individual level, the model reduces the tendency to overestimate predicted LVSD risk in certain clinical conditions, thereby allowing a single validation-derived decision threshold to retain consistent meaning across heterogeneous patient populations and mitigating excessive false-positive predictions when applied uniformly.

However, this benefit comes with the requirement of actual LVEF measurements as reference values, necessitating echocardiography performed by experts, which may limit the widespread applicability of this model. To extend the utility of our approach, we developed a second recalibrated model masked to the LVEF, which also demonstrated subtle performance gains compared to the uncalibrated model. This model operates solely on serial ECG data and does not require LVEF measurements from echocardiography for inference, potentially broadening its applicability to settings where access to echocardiography is limited.

### Model Pretraining for Classification Performance

Pretraining has become a standard approach for enhancing deep learning models, particularly when large datasets are accessible. This strategy has been extensively explored in the AI-ECG field using various data formats and pretraining objectives [27-31]. In this study, pretraining was implemented using a transfer learning [32,33] framework, leveraging labels that were directly extracted from ECG analysis systems. These include automated diagnostic statements and waveform-derived features, which eliminate the need for labor-intensive manual annotations. During pretraining, the model was tasked with predicting these multiple automatically generated labels, enabling it to learn broadly relevant granular ECG representations.

In the subsequent supervised fine-tuning stage, these low-level features were refined while training on a smaller outcome-specific dataset. This is in contrast to training from scratch, in which models often fail to capture subtle or infrequent ECG patterns owing to the limited volume of supervised learning. As a result of incorporating pretraining, our model demonstrated improved performance, with an internal AUROC increase of 0.014 and an external AUROC increase of 0.010, compared to models trained solely on the fine-tuning set.

The capacity for pretraining to use massive datasets should be emphasized. In this study, the pretraining phase encompassed 397,644 ECGs from 191,121 patients, which was nearly 10 times the size of the fine-tuning set (31,099 ECGs from 31,099 patients). This approach allows the model to benefit from large-scale data, even if the pretraining labels are noisy or imprecise, or in certain pretraining methods that are entirely unlabeled. Hence, pretraining enables the model to develop a more generalizable feature extractor that can improve downstream performance across various clinical populations.

### LVSD Detection and AI-ECG

LVSD remains a critical precursor of overt HF and is associated with significant morbidity and mortality. Early detection facilitates the timely initiation of guideline-directed therapy and improves patient outcomes [3]. However, the high resource intensity of echocardiography precludes its use as a universal screening tool. AI-ECG models, such as those developed in this study, present a scalable, low-cost alternative suitable for broader implementation. Our model achieved a strong performance in both standard (12-lead) and reduced-lead configurations, further supporting its adaptability to various ECG acquisition modalities, including emerging handheld and wearable devices.

Our study expands the application of AI-ECG from a screening tool to a longitudinal monitoring tool for at-risk populations. Our model achieved an enhanced diagnostic performance compared to previous studies in at-risk patient groups, such as those with AFIB. In an external validation study of the AI-based identification of LVSD, König et al [14] have reported an AUROC of 0.815 for the AFIB subgroup, which was lower than that of the general cohort. Similarly, in a study by Harmon et al [10], the AUROC for the AFIB subgroup decreased to 0.826, suggesting that separate algorithms may be required for patients with marked electrical abnormalities. Our study provides evidence that our approaches may yield improved diagnostic accuracy in such subgroups [10]. Moreover, sensitivity and specificity also showed minimal variation regardless of comorbid conditions, potentially expanding the use of AI-ECG–based LVSD detection to clinical presentations with a higher burden of comorbidities.

Cardiac function evaluation is important for the timely detection and management of subclinical or progressive cardiac dysfunction. A 2019 report on the appropriate imaging criteria for the assessment of cardiac function in nonvalvular heart disease [34] approved the use of TTE in the routine evaluation of systemic hypertension and the reevaluation of patients previously or currently undergoing cardiotoxic therapy. Furthermore, the recent 2023 Update on European Society of Cardiology Heart Failure Guidelines [35] and analysis from the STRONG-HF [36] trial highlight the importance of frequent and careful follow-up during the intensive titration of medical therapy in the postdischarge period for patients with acute HF. In this context, our AI-ECG algorithm may serve not only as a screening tool to identify cardiac dysfunction but also as a longitudinal monitoring tool to detect signs of clinical deterioration during follow-up in individual patients.

### Clinical Integration and Future Directions

From a clinical perspective, the proposed DeepECG LVSD model could be integrated into routine practice as a background screening or decision-support tool embedded within standard ECG workflows. Similar to prior AI-ECG implementations, the model could operate silently at the time of ECG acquisition, flagging patients at elevated risk for LVSD and prompting targeted referral for confirmatory echocardiography. Such an approach may be particularly valuable in outpatient settings or among patients with multiple comorbidities, where LVSD is frequently underdiagnosed. Nevertheless, several barriers to real-world adoption remain. Workflow integration will require careful consideration to minimize alert fatigue and ensure that model outputs are presented in a clinically actionable manner. Future work should focus on prospective clinical studies to evaluate the impact of AI-assisted ECG screening on downstream testing, clinical decision-making, and patient outcomes. Additional research is also needed to optimize operating thresholds tailored to specific clinical use cases and to assess regulatory, cost, and reimbursement considerations.

### Limitations

This study had some limitations. First, this study was conducted in only 2 tertiary care centers in Korea; further external validation in more diverse populations is essential to extend its generalizability. Second, its retrospective design, along with the use of paired ECG and echocardiography data, may limit generalizability and introduce potential selection bias, highlighting the need for future prospective studies to establish real-world utility and workflow integration. Third, this study did not evaluate the association between the AI model outputs and clinical outcomes. Although the model demonstrated strong technical performance, its prognostic value and clinical utility remain to be validated in real-world patient outcomes. Fourth, although our recalibration method empirically demonstrated benefits in the model performance, we were unable to address the specific considerations related to the best implementation. The selection of the model architecture and optimal format of historical data as auxiliary inputs remains an open research question. Also, although the masked-LVEF approach reduces reliance on prior echocardiographic data, it remains an early-stage methodology with limited clinical validation. Fifth, ECGs and TTE measurements were not obtained simultaneously, and the temporal gap between acquisitions may introduce inaccuracies in the performance metrics. Prospective data collection using simultaneous ECG and TTE measurements may provide more reliable assessments in future studies. Finally, as with many AI-based models, the algorithm operates as a “black box” that limits the interpretability and transparent understanding of the decision-making process.

### Conclusion

The AI-ECG model for LVSD detection demonstrated a strong performance through pretraining on a large dataset and a novel recalibration approach. By addressing both diagnostic performance and real-world clinical applications, our work pursues the practical integration of AI-driven diagnostics into cardiovascular screening and highlights the need for future prospective, randomized studies to confirm the clinical benefits.

## Supplementary material

10.2196/83127 Multimedia Appendix 1 Diagnostic performance metrics (sensitivity, specificity, positive predictive value, and negative predictive value) of DeepECG models at fixed thresholds.

## Abbreviations

AFIB: atrial fibrillation
AI: artificial intelligence
AI-ECG: artificial intelligence-augmented electrocardiography
AUROC: area under the receiver operating curve
ECG: electrocardiography
HF: heart failure
*ICD-10*: *International Statistical Classification of Diseases, Tenth Revision*
LBBB: left bundle branch block
LVEF: left ventricular ejection fraction
LVSD: left ventricular systolic dysfunction
NPV: negative predictive value
PPV: positive predictive value
TTE: transthoracic echocardiography
TTE-ECG: transthoracic echocardiography-electrocardiogram
