# Supplementary material for: Deep Learning Model Using Transfer Learning for Detecting Left Ventricular Systolic Dysfunction: Retrospective Algorithm Development and Validation Study
Source: JMIR Med Inform. 2026 Apr 24;14:e83127. doi: 10.2196/83127 (PMC13108837; doi:10.2196/83127)
Supplement: Multimedia Appendix 1 [file medinform-v14-e83127-s001.docx]

**Deep Learning Model using Transfer Learning for Detecting Left Ventricular Systolic Dysfunction: Algorithm Development and Validation**

Sungjae Lee, MS^b,†^, Jung-Woo Son, MD^a,^^†^, Sung-Ai Kim, MD, PhD^c,†^, Min-Soo Ahn MD, PhD^a^, Sang Jun Lee, MD^a^, Sang-Jin Han, MD, PhD^c^, Taehyun Joo, MD^b^, Yeongyeon Na, MS^b^, Sunghoon Joo, PhD^b^, Hyun Jin Ahn, MD^b^, Mineok Chang, MD, PhD^b^, Yeha Lee, PhD^b^, Young Jun Park, MD^a,*^

^a^Division of Cardiology, Department of Internal Medicine, Wonju Severance Christian Hospital, Yonsei University Wonju College of Medicine, Wonju, Republic of Korea

^b^VUNO Inc., Seoul, Republic of Korea

^c^Division of Cardiology, Department of Internal Medicine, Hallym Sacred Heart Hospital, Hallym University College of Medicine, Anyang, Republic of Korea

**Table S1. Sensitivity****, PPV, and NPV at a fixed** **threshold with specificity of 0.90****, evaluated with 12-lead, external, and follow-up TTE-ECG** **pairs (prevalence: 13.88%).**

| Configuration | AUROC | Sens. | Spec. | PPV | NPV | Threshold |
| --- | --- | --- | --- | --- | --- | --- |
| DeepECG LVSD | 0.910 | 0.713 | 0.900 | 0.535 | 0.951 | 0.937 |
| DeepECG LVSD (recalibrated,  masked to LVEF) | 0.920 | 0.752 | 0.900 | 0.548 | 0.958 | 0.569 |
| DeepECG (recalibrated) | 0.940 | 0.824 | 0.900 | 0.569 | 0.969 | 0.438 |

Abbreviations: AUROC, area under the receiver-operating-characteristic curve; Sens., sensitivity; Spec., specificity, PPV, positive predictive value; NPV, negative predictive value

**Table S2. Specificity, PPV, and NPV at a fixed threshold with sensitivity of 0.90, evaluated with 12-lead, external, and follow-up TTE-ECG pairs (prevalence: 13.88%).**

| Configuration | AUROC | Sens. | Spec. | PPV | NPV | Threshold |
| --- | --- | --- | --- | --- | --- | --- |
| DeepECG LVSD | 0.910 | 0.900 | 0.757 | 0.373 | 0.979 | 0.630 |
| DeepECG LVSD (recalibrated,  masked to LVEF) | 0.920 | 0.900 | 0.793 | 0.412 | 0.980 | 0.313 |
| DeepECG (recalibrated) | 0.940 | 0.900 | 0.830 | 0.461 | 0.981 | 0.276 |

Abbreviations: AUROC, area under the receiver-operating-characteristic curve; Sens., sensitivity; Spec., specificity, PPV, positive predictive value; NPV, negative predictive value
